# Practical Points

**Published:** 1908-12-05

**Authors:** 


### PRACTICAL POINTS.

**Dry Pleurisy.**

Cases of dry pleurisy require very little treatment except some counter-irritation, a diaphoretic, a purgative, and perhaps a sedative to relieve pain, or some strapping of the chest to limit the amount of movement.—*Sir James Barr.*

**Graves's Disease.**

I consider the diet is an important part of the treatment, and I recommend for most cases plenty of fruit, vegetables, cream, bread, butter, eggs, and very little meat. Further, patients with this malady cannot have too much fresh air.—*Dr. Hector Mackenzie.*

